# Supplementary material for: Structural insights into ubiquitin recognition and Ufd1 interaction of Npl4
Source: Nat Commun. 2019 Dec 13;10:5708. doi: 10.1038/s41467-019-13697-y (PMC6910952; doi:10.1038/s41467-019-13697-y)
Supplement: Supplementary file 1 — Supplementary Information [file 41467_2019_13697_MOESM1_ESM.pdf]

Supplementary Information

Structural insights into ubiquitin recognition and Ufd1 interaction of Npl4

Sato et al.

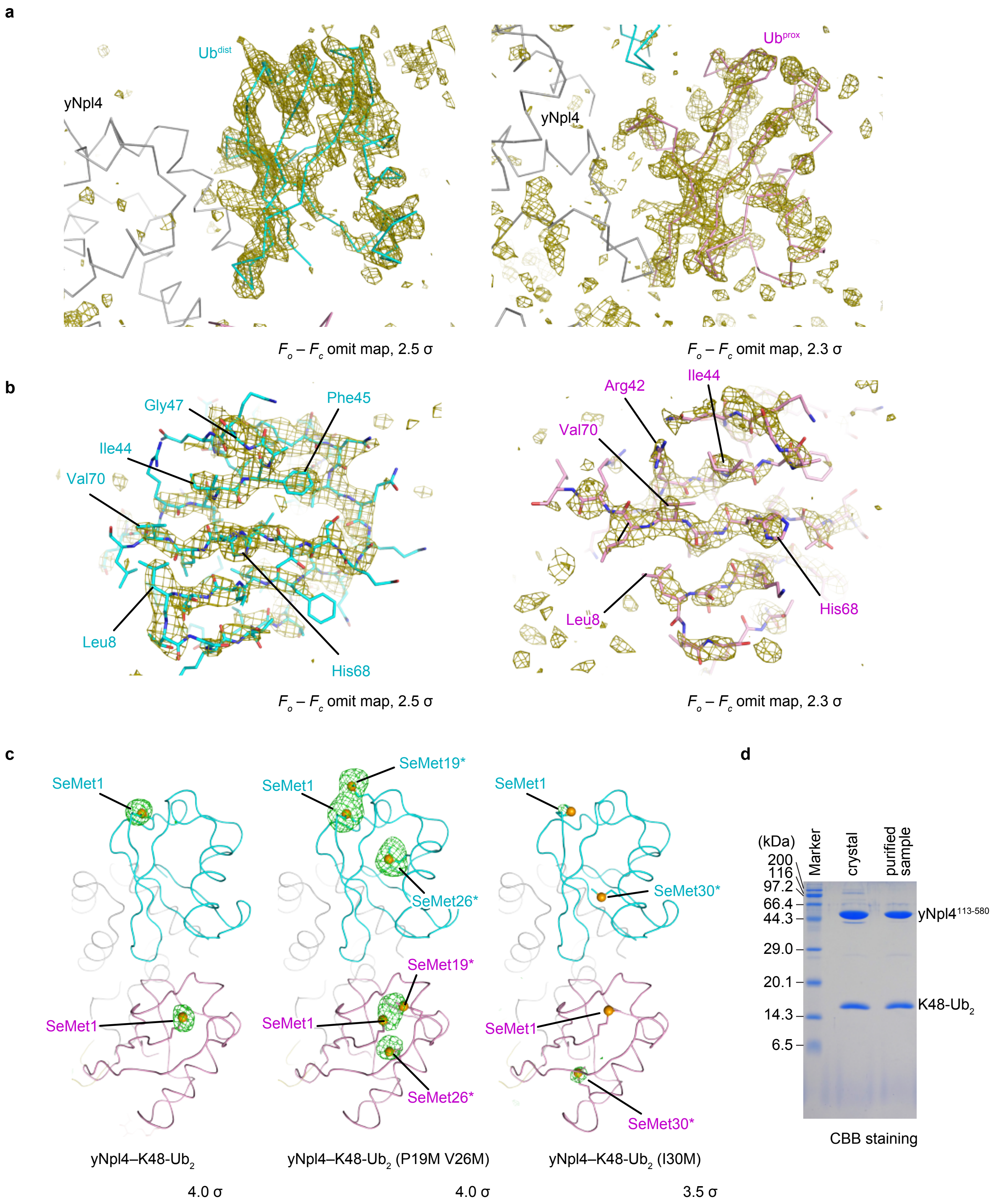

Supplementary Figure 1 Additional information of crystallography.

The coloring scheme is the same as that in Fig. 2a.

(a) F_o–F_c omit maps of Ub^{dist} (left, cyan) and Ub^{prox} (right, pink). The maps were calculated with Ub^{dist} removed (left) and with Ub^{prox} removed (right), and are shown as olive mesh contoured at 2.5 and 2.3 σ levels, respectively.

(b) Close-up views of (a). The yNpl4-interacting sites of Ub^{dist} (left) and Ub^{prox} (right) are magnified.

(c) Anomalous difference Fourier maps of the SeMet-labeled K48-Ub$_2$ (WT, left; P19M V26M, middle; I30M, right). Density maps are shown as green mesh (contoured at 4.0 σ level for WT and P19M V26M and 3.5 σ level for I30M).

(d) SDS-PAGE analysis of yNpl4–K48-Ub$_2$ crystals with Coomassie brilliant blue staining. The crystals were washed with the mother liquor before SDS-PAGE. Source data are provided as a Source Data file.

a

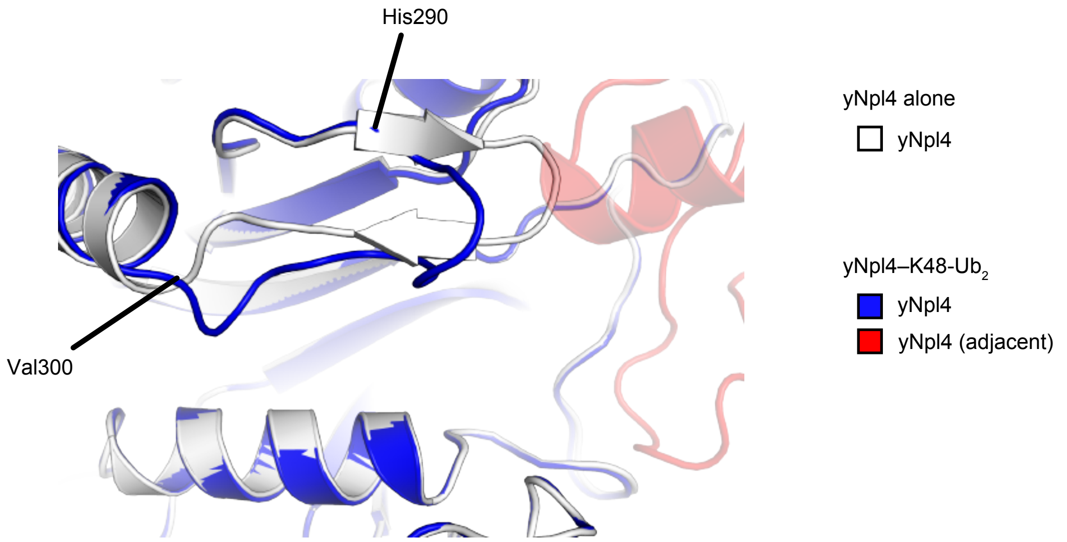

b

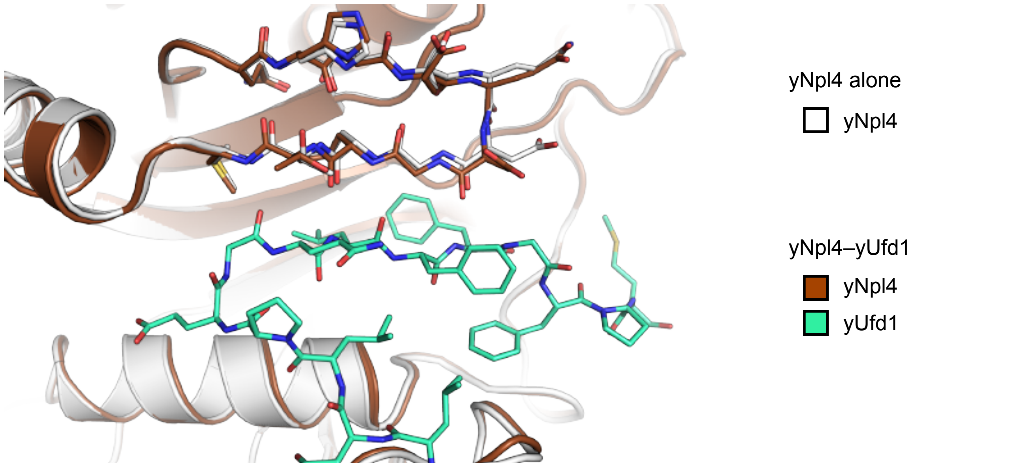

Supplementary Figure 2 Conformational differences in Ins-1 of yNpl4 between the apo and K48-Ub$_2$-bound states and between the apo and Ufd1-bound states.
(a) Superposition of the apo and K48-Ub$_2$-bound yNpl4 structures. The adjacent yNpl4 molecule in the asymmetric unit of the yNpl4–K48-Ub$_2$ crystal is also shown.
(b) Superposition of the apo and yUfd1-bound yNpl4 structures.

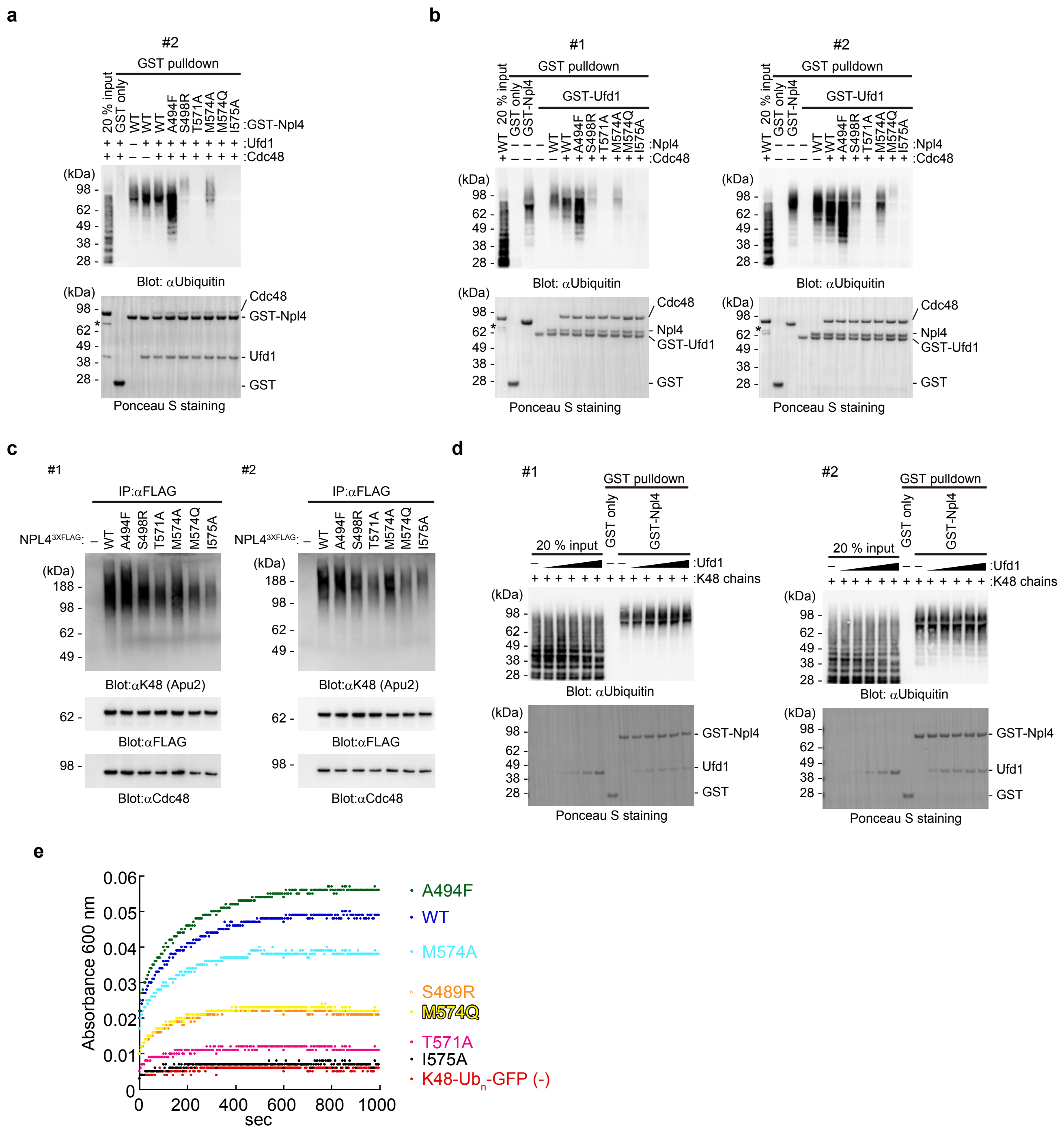

Supplementary Figure 3 Analyses of the mutations of the K48 chain-binding site of yNpl4.
(a) A repeat of Fig. 2d with distinct samples.
(b) Analysis of Ub chain binding of yNpl4, yUfd1, the UN heterodimer, and the yCdc48–UN complex with indicated mutations by pulldown assays. GST-yNpl4 (lane 3) and GST-yUfd1 (lane 4-12) were used. The bound Ub chains were detected by immunoblotting with anti-Ub antibody (upper panel). Blot membranes were stained with Ponceau S (lower panel). 20% input means 20% of the volume of the sample (K48 chain, Cdc48 and yNpl4) that was mixed with the GST-yNpl4 bound glutathione resin. Asterisks indicate contamination. Experiments were performed twice with distinct samples with similar results.
(c) Analysis of K48 chain binding of yNpl4 with indicated mutations in vivo. Wild-type or mutant yNpl4-3xFLAG was expressed in *npl4Δ* cells. yNpl4-3xFLAG was immunoprecipitated with anti-FLAG antibody, and was subjected to immunoblotting with the indicated antibodies. Experiments were performed twice with distinct samples with similar results.
(d) Analysis of K48 chain binding of yNpl4 with the increasing concentrations of yUfd1 (0.25–4 μM). The bound K48 chains were detected by immunoblotting with anti-Ub antibody (upper panel). Blot membranes were stained with Ponceau S (lower panel). 20% input means 20% of the volume of the sample (K48 chain and yUfd1) that was mixed with the GST-Npl4-bound glutathione resin. Experiments were performed twice with distinct samples with similar results.
(e) *In vitro* ATPase assays of Cdc48-UN with indicated mutations of yNpl4. The polyubiquitylated GFP (Ub_n-GFP) was incubated with Cdc48 and UN heterodimer. After addition of ATP and BIOMOL Green, the absorption was followed over time. One out of ten technical replicates is shown.
(a-d) Source data are provided as a Source Data file.

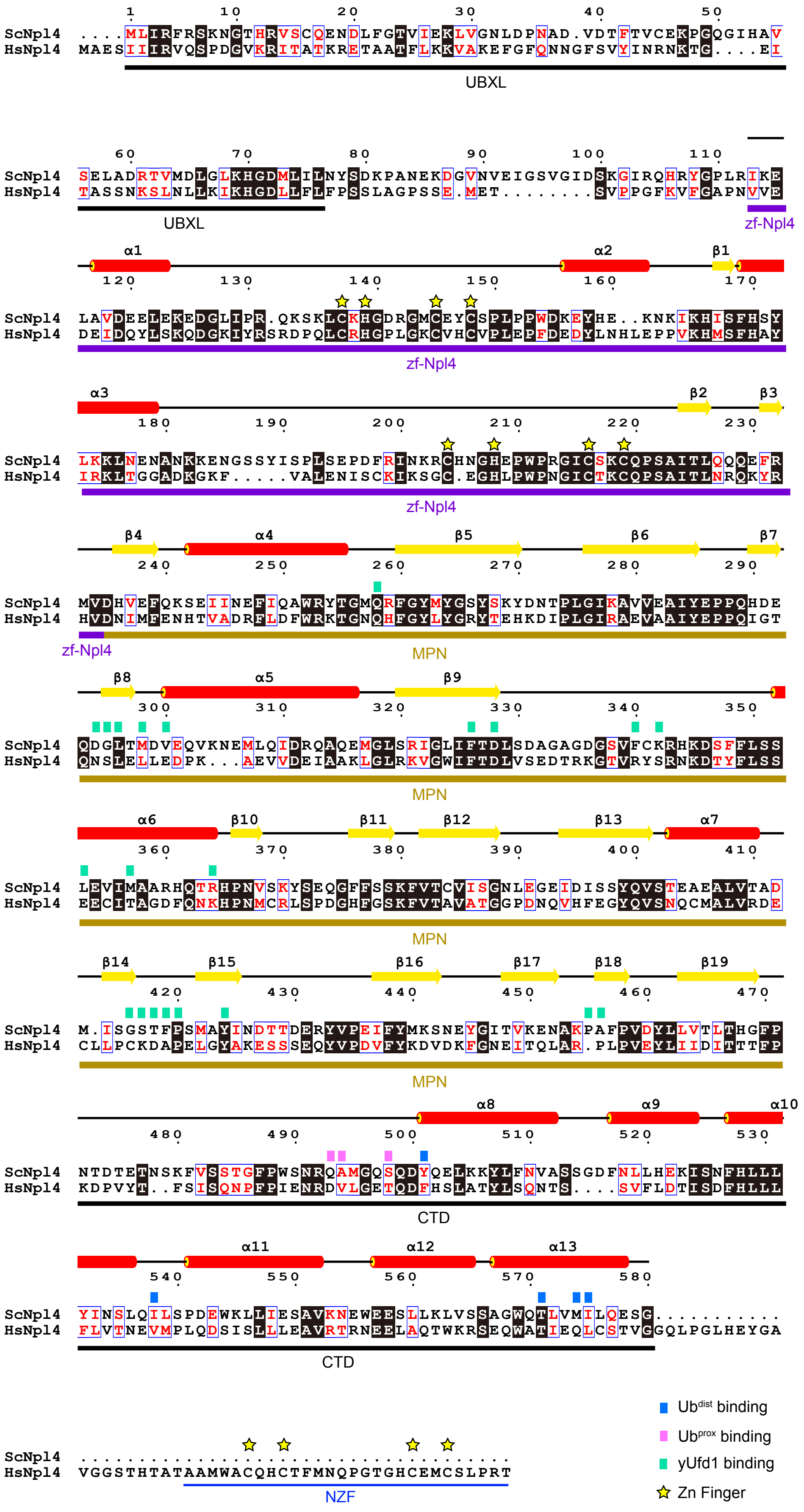

Supplementary Figure 4 Amino-acid sequence alignment of yNpl4 and hNpl4.
Conserved residues are colored white with black backgrounds, whereas residues with similar physicochemical properties are colored red. The Ub^{dist}-, Ub^{prox}-, and yUfd1-interacting residues of yNpl4 are indicated by blue, purple, and turquoise rectangles, respectively. The Zn^{2+}-coordinating residues are indicated by yellow stars. The residue number and secondary structure of yNpl4 are shown above the alignment.

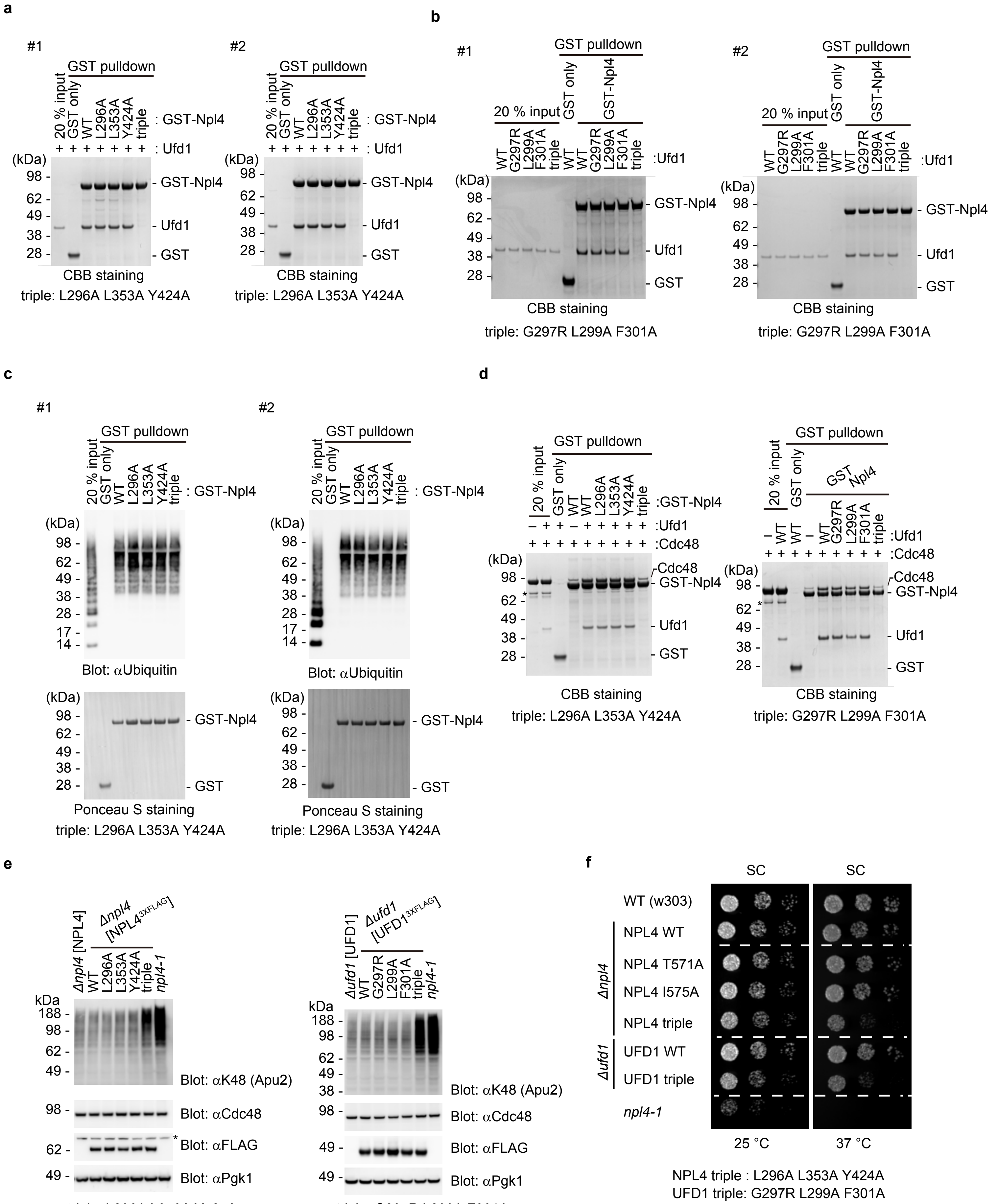

a
#1
GST pulldown
20 % input
GST only
WT
L296A
L353A
Y424A
triple
: GST-Npl4
: Ufd1
(kDa)
98
62
49
38
28
GST-Npl4
Ufd1
GST
CBB staining
triple: L296A L353A Y424A
#2
b
#1
GST pulldown
20 % input
GST only
GST-Npl4
WT
G297R
L299A
F301A
triple
:Ufd1
CBB staining
triple: G297R L299A F301A
#2
c
#1
Blot: αUbiquitin
Ponceau S staining
17
14
#2
d
:GST-Npl4
:Ufd1
:Cdc48
Cdc48
GST-Npl4
Ufd1
GST
e
Δnpl4 [NPL4]
Δnpl4 [NPL4^3XFLAG]
npl4-1
kDa
188
Blot: αK48 (Apu2)
Blot: αCdc48
Blot: αFLAG
Blot: αPgk1
Δufd1 [UFD1]
Δufd1 [UFD1^3XFLAG]
triple: G297R L299A F301A
f
SC
WT (w303)
Δnpl4
NPL4 WT
NPL4 T571A
NPL4 I575A
NPL4 triple
Δufd1
UFD1 WT
UFD1 triple
npl4-1
25 °C
37 °C
NPL4 triple : L296A L353A Y424A
UFD1 triple: G297R L299A F301A

Supplementary Figure 5 Mutational analyses of the yNpl4–yUfd1 interaction site.
(a, b) Analysis of the binding between GST-yNpl4 and yUfd1 by pulldown assays. The results from yNpl4 mutants (a) and yUfd1 mutants (b) are shown. The bound proteins were analyzed by SDS-PAGE and stained with Coomassie brilliant blue. 20% input means 20% of the volume of the sample (yUfd1) that was mixed with the GST-yNpl4-bound glutathione resin. Experiments were performed twice with distinct samples with similar results.
(c) Analysis of Ub chain binding of yNpl4 with indicated mutations by pulldown assays. The bound Ub chains were detected by immunoblotting with anti-Ub antibody (upper panel). Blot membranes were stained with Ponceau S (lower panel). 20% input means 20% of the volume of the sample (K48 chain) that was mixed with the GST-Npl4-bound glutathione resin. Experiments were performed twice with distinct samples with similar results.
(d) Analysis of the binding between GST-yNpl4 and yUfd1–Cdc48 by pulldown assay. The results from yNpl4 mutants (left) or yUfd1 mutants (right) are shown. 20% input means 20% of the volume of the sample (Cdc48 or yUfd1-Cdc48) that was mixed with the GST-yNpl4-bound glutathione resin. Asterisks indicate contamination. The bound proteins were analyzed by SDS-PAGE and stained with Coomassie brilliant blue.
(e) Accumulation of Ub conjugates in the cells expressing wild-type or mutant yUfd1 or yNpl4. Total lysate was subjected to immunoblotting with the indicated antibodies. Anti-Pgk1 was used as a loading control. An asterisk indicates non-specific bands.
(f) Yeast plate test of mutant Npl4-expressing *npl4Δ* cells or mutant Ufd1-expressing *ufd1Δ* cells. The cells were spotted in 1:5 dilutions, and incubated at 25 or 37 °C. The *npl4-1* temperature-sensitive strain was also examined as an Npl4-deficient control.
(a-e) Source data are provided as a Source Data file.

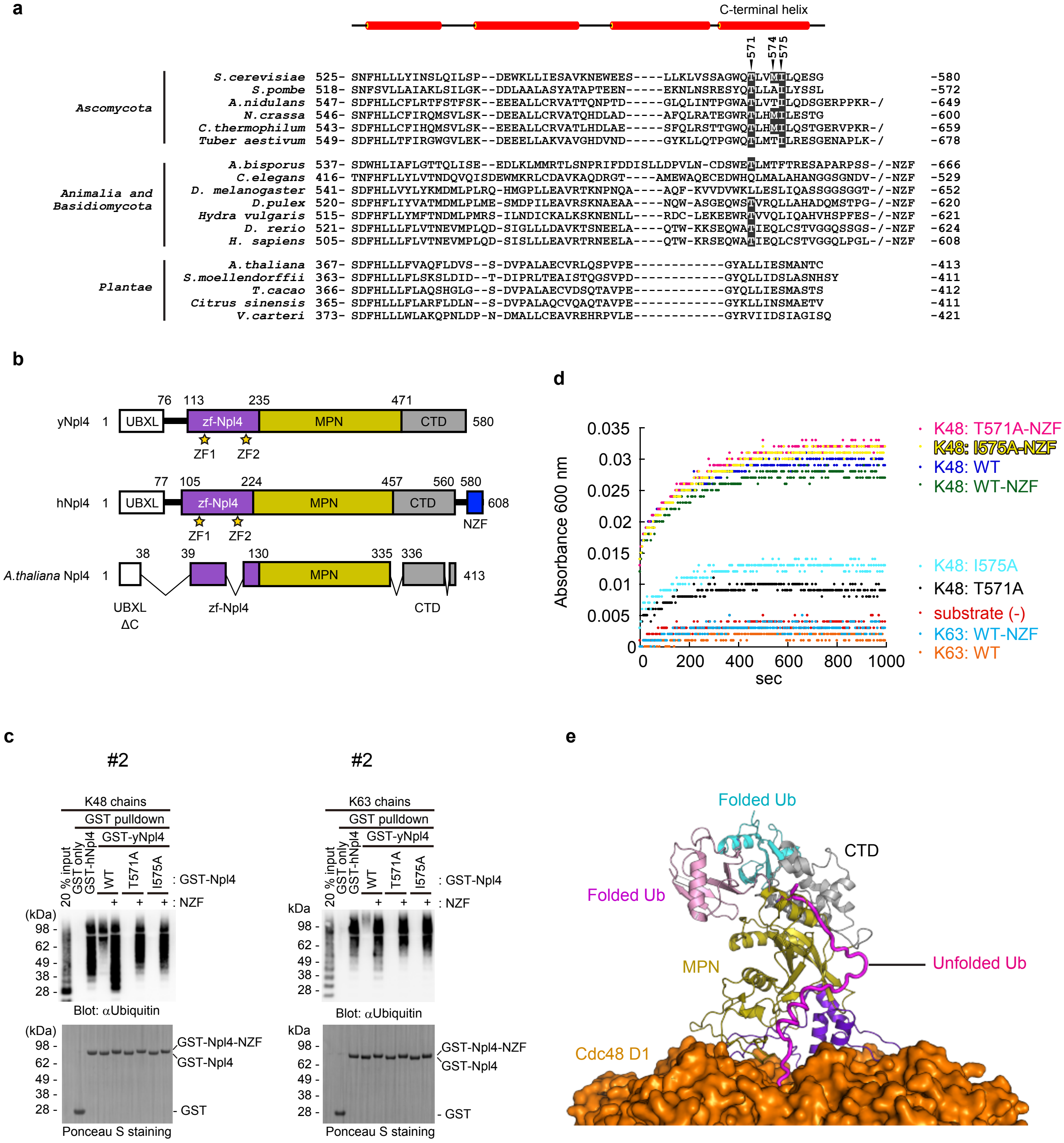

Supplementary Figure 6 Comparison of Npl4 proteins from different organisms.

(a) Amino-acid sequence alignment of Npl4 proteins from *Ascomycota*, *Animalia*, *Basidiomycota*, and *Plantae*.

(b) Domain compositions of Npl4 proteins from yeast, human, and *Arabidopsis*.

(c) Repeats of Fig. 4a (left) and Fig. 4b (right) with distinct samples. Source data are provided as a Source Data file.

(d) *In vitro* ATPase assays of Cdc48-UN with indicated variants of Ufd1 or Ub chains. The polyubiquitylated GFP (Ub_n-GFP) was incubated with Cdc48 and UN heterodimer or Ufd1–Npl4-NZF heterodimer. After addition of ATP and BIOMOL Green, the absorption was followed over time. One out of three technical replicates is shown.

(e) Close-up view of the area around the Npl4 and Ub moieties in cryo-EM structure of the Cdc48–UN complex (PDB 6OA9)[22]. The coloring scheme is the same as that in Fig. 1. The folded Ub moieties corresponding to Ub^{dist} and Ub^{prox} are colored cyan and pink, respectively. The unfolded Ub is colored magenta.

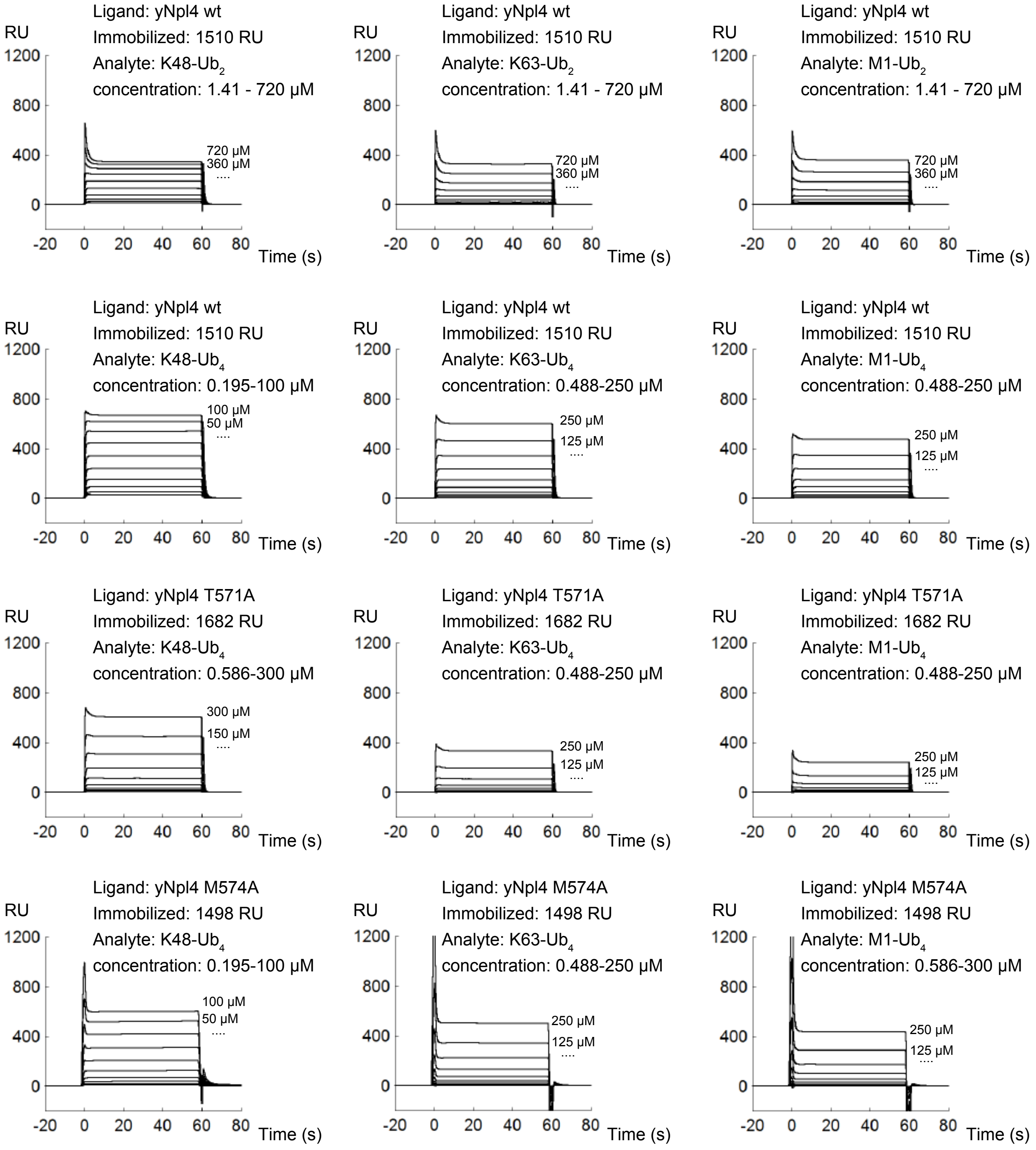

Supplementary Figure 7 (continued)

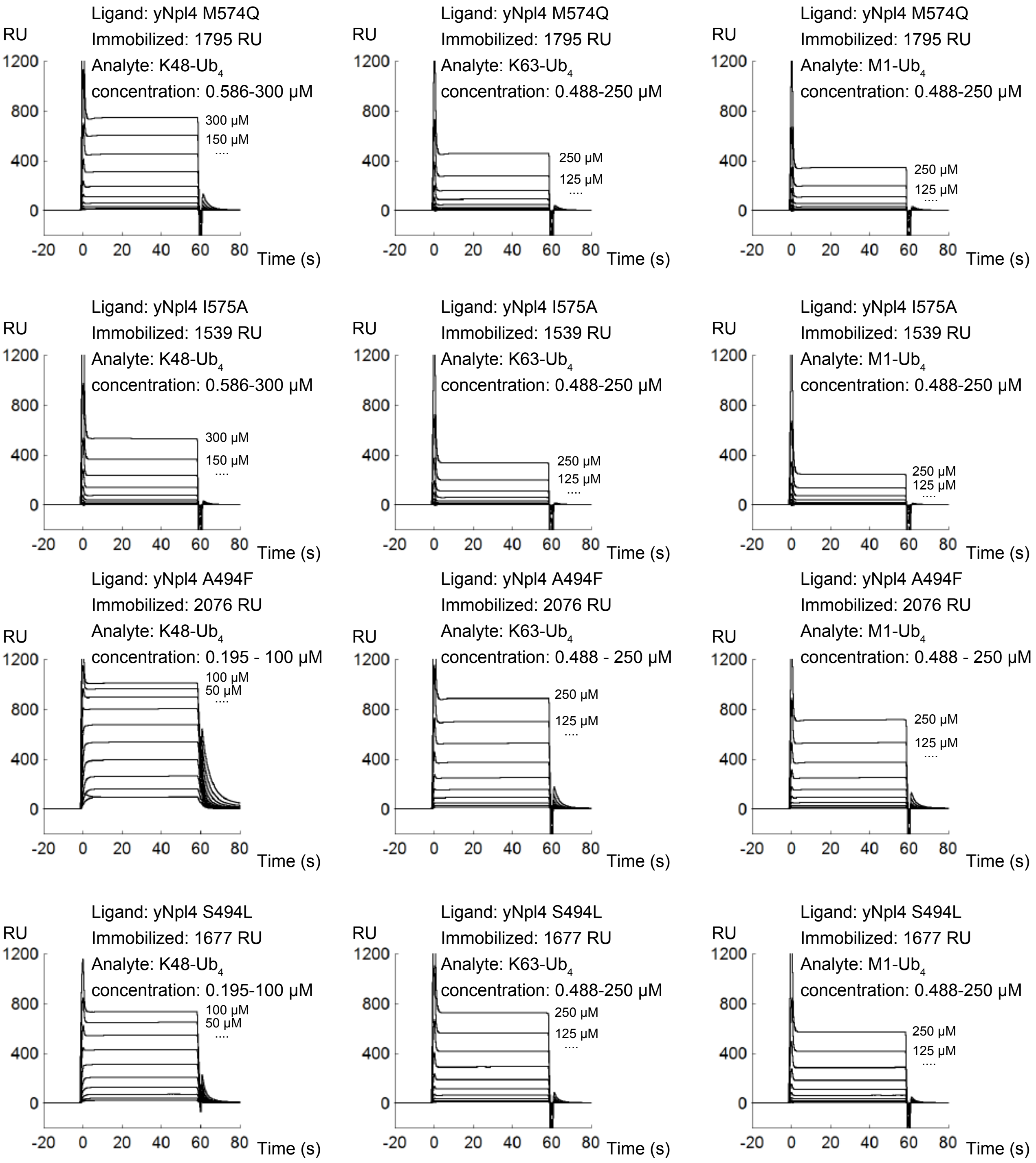

Supplementary Figure 7 (continued)

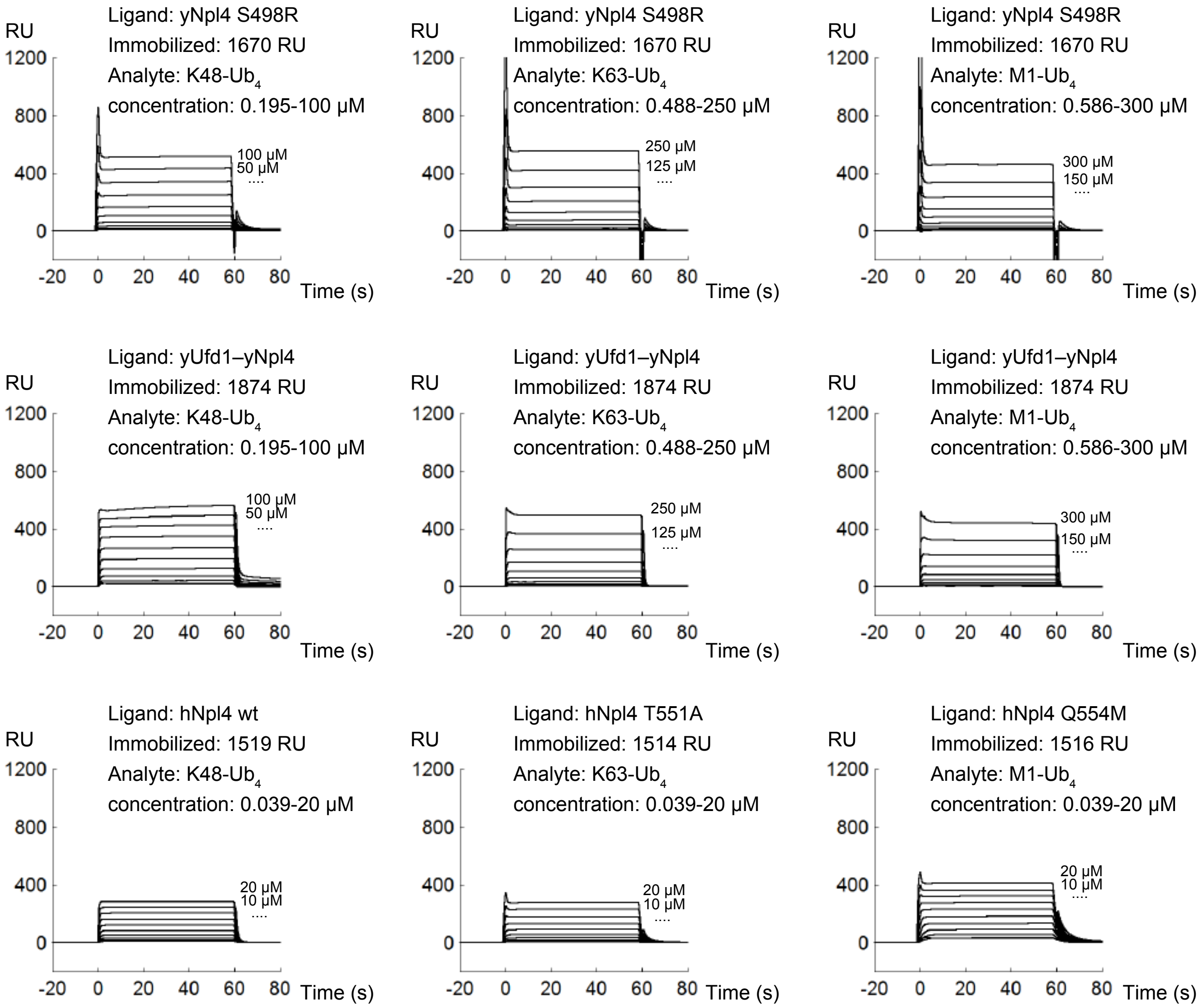

Supplementary Figure 7 SPR sensorgrams that were used to calculate the affinities shown in Table 2. One representative sensorgram from three independent measurements is shown for each ligand–analyte pair.

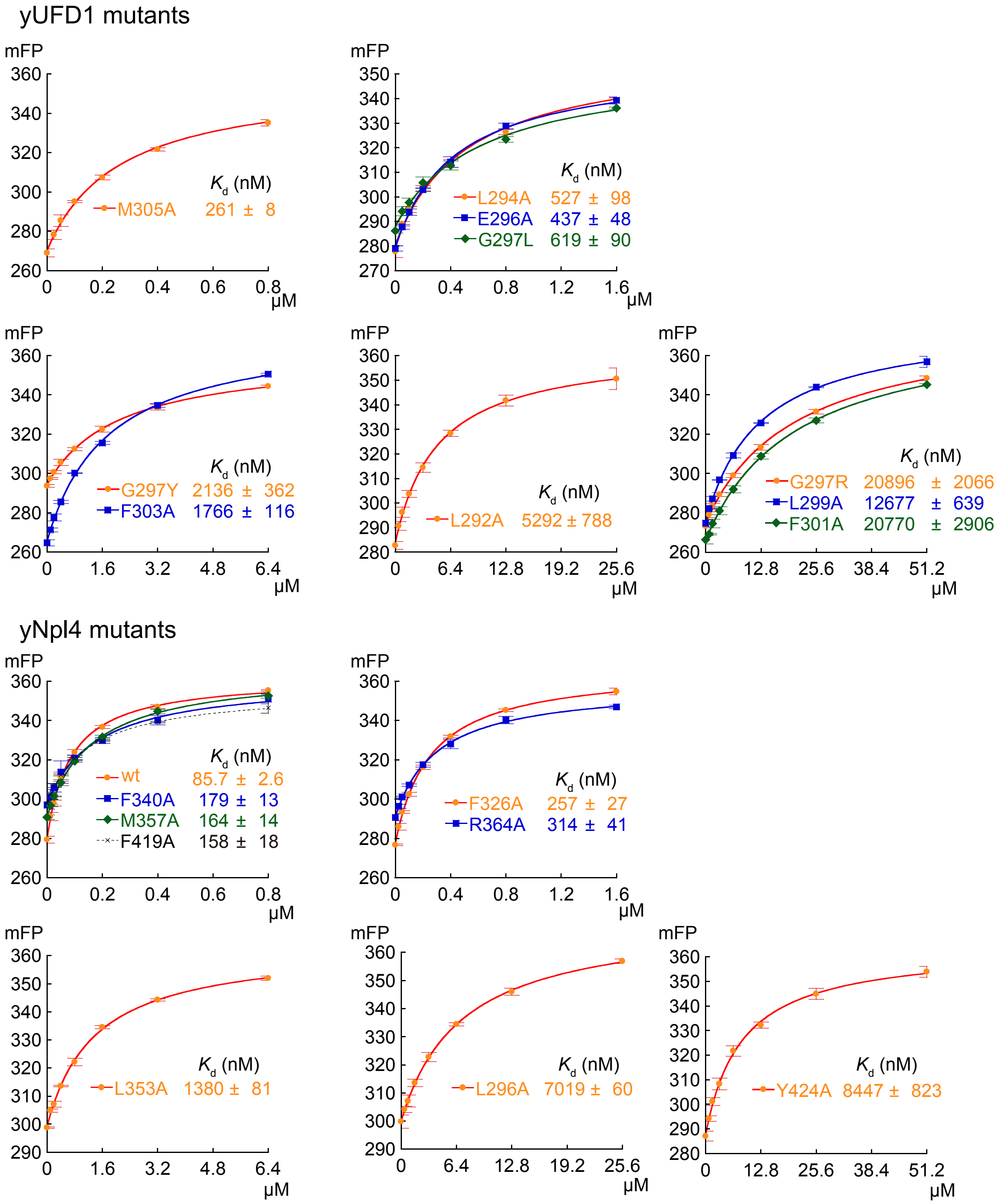

Supplementary Figure 8 Fluorescence anisotropy-based affinity measurements of yNpl4 or yUfd1 mutants, related to Table 3.

Fluorescence polarization values are plotted as a function of the concentration of yNpl4. Error bars represent the standard deviations from the mean values of measurements performed in triplicate. The data were fitted to a one-site binding model to derive dissociation constants (K_d). Data are presented as mean ± standard deviation.

Supplementary Table 1. Oligonucleotide primers used in this study.

Primer #	Primer Sequence (5' to 3')	Description
1	CGCGCGCCATATGATCAAGGAATTGGCG GTAG	forward primer to clone yNpl4 (113-580) into pET28a vector
2	GCGCGGATCCATCAAGGAATTGGCGGTA G	forward primer to clone yNpl4 (113-580) into pGEX6P1 vector
3	GCGCCTCGAGTTAGCCGGATTCCTGAAG GATC	reverse primer to clone yNpl4 (113-580) into pGEX6P1 vector and pET28a vector
4	TAGATGAGGAACTCGCAGCAGCGGACG GGTTGATCCC	forward primer to mutate yNpl4 Glu123, Lys124 and Glu125 to Ala-Ala-Ala
5	GGGATCAACCCGTCCGCTGCTGCGAGTT CCTCATCTA	reverse primer to mutate yNpl4 Glu123, Lys124 and Glu125 to Ala-Ala-Ala
6	AGCAAGATGGAGCAACGATGGATG	forward primer to mutate yNpl4 Leu296 to Alanine
7	CATCCATCGTTGCTCCATCTTGCT	reverse primer to mutate yNpl4 Leu296 to Alanine
8	TTGGGTTGATCGCCACCGATCTGT	forward primer to mutate yNpl4 Phe326 to Alanine
9	ACAGATCGGTGGCGATCAACCCAA	reverse primer to mutate yNpl4 Phe326 to Alanine
10	TCCTGAGCTCAGCGGAAGTGATAA	forward primer to mutate yNpl4 Leu353 to Alanine
11	TTATCACTTCCGCTGAGCTCAGGA	reverse primer to mutate yNpl4 Leu353 to Alanine
12	TGGAAGTGATAGCGGCTGCTCGTC	forward primer to mutate yNpl4 Met357 to Alanine
13	GACGAGCAGCCGCTATCACTTCCA	reverse primer to mutate yNpl4 Met357 to Alanine
14	GTCACCAAACCGCTCATCCGAATG	forward primer to mutate yNpl4 Arg364 to Alanine
15	CATTCGGATGAGCGGTTTGGTGAC	reverse primer to mutate yNpl4 Arg364 to Alanine
16	CGGGTTCAACCGCTCCCAGCATGG	forward primer to mutate yNpl4 Phe419 to Alanine
17	CCATGCTGGGAGCGGTTGAACCCG	reverse primer to mutate yNpl4 Phe419 to Alanine
18	CCAGCATGGCGGCTATTAACGACA	forward primer to mutate yNpl4 Tyr424 to Alanine
19	TGTCGTTAATAGCCGCCATGCTGG	reverse primer to mutate yNpl4 Tyr424 to Alanine
20	GCAACCGCCAATTTATGGGCCAAAG	forward primer to mutate yNpl4 Ala494 to Phenylalanine
21	CTTTGGCCCATAAATTGGCGGTTGC	reverse primer to mutate yNpl4 Ala494 to Phenylalanine
22	CGATGGGCCAACTTCAGGACTATC	forward primer to mutate yNpl4 Ser498 to Leucine
23	GATAGTCCTGAAGTTGGCCCATCG	reverse primer to mutate yNpl4 Ser498 to Leucine
24	ATGGGCCAAAGACAGGACTATCA	forward primer to mutate yNpl4 Ser498 to Arginine
25	TGATAGTCCTGTCTTTGGCCCAT	reverse primer to mutate yNpl4 Ser498 to Arginine
26	CAGGTTGGCAGGCACTGGTCATG	forward primer to mutate yNpl4 Thr571 to Alanine
27	CATGACCAGTGCCTGCCAACCTG	reverse primer to mutate yNpl4 Thr571 to Alanine
28	AGACACTGGTCGCGATCCTTCAGG	forward primer to mutate yNpl4 Met574 to Alanine
29	CCTGAAGGATCGCGACCAGTGTCT	reverse primer to mutate yNpl4 Met574 to Alanine
30	AGACACTGGTCCAGATCCTTCAGG	forward primer to mutate yNpl4 Met574 to Glutamine
31	CCTGAAGGATCTGGACCAGTGTCT	reverse primer to mutate yNpl4 Met574 to Glutamine
32	CACTGGTCATGGCCCTTCAGGAAT	forward primer to mutate yNpl4 Ile575 to Alanine
33	ATTCCTGAAGGGCCATGACCAGTG	reverse primer to mutate yNpl4 Ile575 to Alanine
34	CGCGCGCCATATGGAACCGGCCAAACTT GATT	forward primer to clone yUfd1 (288-305) into pCold-GST vector and pCold-SUMO vector
35	GCGCCTCGAGTTACATAGGAAAGCCAAA GAAC	reverse primer to clone yUfd1 (288-305) into pCold-GST vector
36	GCGCCTCGAGTCAGCAACAACCAGGGC AACACCAACCCATAGGAAAGCCAAAGAA CAGT	reverse primer to clone yUfd1 (288-305) with the C-terminal GWCCPGCC sequence into pCold-SUMO vector
37	AACCGGCCAAAGCTGATTTACCTGAGGG TCAAC	forward primer to mutate yUfd1 Leu292 to Alanine
38	CAGGTAAATCAGCTTTGGCCGGTTCCATA TGAG	reverse primer to mutate yUfd1 Leu292 to Alanine
39	CCAAACTTGATGCACCTGAGGGTC	forward primer to mutate yUfd1 Leu294 to Alanine
40	GACCCTCAGGTGCATCAAGTTTGG	reverse primer to mutate yUfd1 Leu294 to Alanine
41	TGATTTACCTGCGGGTCAACTGT	forward primer to mutate yUfd1 Glu296 to Alanine
42	ACAGTTGACCCGCAGGTAAATCA	reverse primer to mutate yUfd1 Glu296 to Alanine
43	ATTTACCTGAGCTTCAACTGTTCT	forward primer to mutate yUfd1 Gly297 to Leucine
44	AGAACAGTTGAAGCTCAGGTAAAT	reverse primer to mutate yUfd1 Gly297 to Leucine
45	ATTTACCTGAGCGTCAACTGTTC	forward primer to mutate yUfd1 Gly297 to Arginine

46	GAACAGTTGACGCTCAGGTAAAT	reverse primer to mutate yUfd1 Gly297 to Arginine
47	ATTTACCTGAGTATCAACTGTTCT	forward primer to mutate yUfd1 Gly297 to Tyrosine
48	AGAACAGTTGATACTCAGGTAAAT	reverse primer to mutate yUfd1 Gly297 to Tyrosine
49	CTGAGGGTCAAGCGTTCTTTGGCT	forward primer to mutate yUfd1 Leu299 to Alanine
50	AGCCAAAGAACGCTTGACCCTCAG	reverse primer to mutate yUfd1 Leu299 to Alanine
51	GTCAACTGTTCGCTGGCTTTCCTA	forward primer to mutate yUfd1 Phe301 to Alanine
52	TAGGAAAGCCAGCGAACAGTTGAC	reverse primer to mutate yUfd1 Phe301 to Alanine
53	TGTTCTTTGGCGCTCCTATGGGTT	forward primer to mutate yUfd1 Phe303 to Alanine
54	AACCCATAGGAGCGCCAAAGAACA	reverse primer to mutate yUfd1 Phe303 to Alanine
55	TTGGCTTTCCTGCGGGTTGGTGTT	forward primer to mutate yUfd1 Met305 to Alanine
56	AACACCAACCCGCAGGAAAGCCAA	reverse primer to mutate yUfd1 Met305 to Alanine
57	GATTTACCTGAGCGTCAAGCGTTCTTTGG CTTTCCTATG	forward primer to mutate yUfd1 Gly296 and L299 to Arg-Ala
58	CATAGGAAAGCCAAAGAACGCTTGACGC TCAGGTAAATC	reverse primer to mutate yUfd1 Gly296 and L299 to Arg-Ala
59	GCGTCAAGCGTTCGCTGGCTTTCCTATGG	forward primer to mutate yUfd1 Gly296, L299 t anf F301 to Arg-Ala-Ala
60	CCATAGGAAAGCCAGCGAACGCTTGACG C	reverse primer to mutate yUfd1 Gly296 and L299 to Arg-Ala-Ala
61	TCGAGGTTGAAATGTCGGATACGAT	forward primer to mutate Ub Pro19 to Methionine
62	ATCGTATCCGACATTTCAACCTCGA	reverse primer to mutate Ub Pro19 to Methionine
63	CGATAGAAAATATGAAGGCCAAGAT	forward primer to mutate Ub Val26 to Methionine
64	ATCTTGGCCTTCATATTTTCTATCG	reverse primer to mutate Ub Val26 to Methionine
65	AAGGCCAAGATGCAGGATAAGGA	forward primer to mutate Ub Ile30 to Methionine
66	TCCTTATCCTGCATCTTGGCCTT	reverse primer to mutate Ub Ile30 to Methionine
67	CGCGCGCCATATGGTGGTCGAGGATGAA ATCG	forward primer to clone hNpl4 (105-608) into pET28a vector
68	GCGCCTCGAGTTACGTGCGTGGCAGGCT ACAC	reverse primer to clone hNpl4 (105-608) into pET28a vector
69	AGCAATGGGCTGCCATTGAACAG	forward primer to mutate hNpl4 Thr551 to Alanine
70	CTGTTCAATGGCAGCCCATTGCT	reverse primer to mutate hNpl4 Thr551 to Alanine
71	CTACCATTGAAATGTTATGCAGTA	forward primer to mutate hNpl4 Gln554 to Methionine
72	TACTGCATAACATTTCAATGGTAG	reverse primer to mutate hNpl4 Gln554 to Methionine
73	CCATGGAGCAATCGACAATTTATGGGGC AATCTCAAG	forward primer to mutate yNpl4-3xFLAG Ala494 to Phenylalanine
74	CTTGAGATTGCCCCATAAATTGTCGATTG CTCCATGG	reverse primer to mutate yNpl4-3xFLAG Ala494 to Phenylalanine
75	CGACAAGCTATGGGGCAACGTCAAGATT ATCAAGAAT	forward primer to mutate yNpl4-3xFLAG Ser498 to Arginine
76	ATTCTTGATAATCTTGACGTTGCCCCATAG CTTGTCG	reverse primer to mutate yNpl4-3xFLAG Ser498 to Arginine
77	GGCTGGTTGGCAAGCGTTAGTCATGATC	forward primer to mutate yNpl4-3xFLAG Thr571 to Alanine
78	GATCATGACTAACGCTTGCCAACCAGCC	reverse primer to mutate yNpl4-3xFLAG Thr571 to Alanine
79	GGCAAACGTTAGTCGCGATCCTTCAGGA AAG	forward primer to mutate yNpl4-3xFLAG Met574 to Alanine
80	CTTTCCTGAAGGATCGCGACTAACGTTTG CC	reverse primer to mutate yNpl4-3xFLAG Met574 to Alanine
81	GTTGGCAAACGTTAGTCCAGATCCTTCAG GAAAGC	forward primer to mutate yNpl4-3xFLAG Met574 to Glutamine
82	GCTTTCCTGAAGGATCTGGACTAACGTTT GCCAAC	reverse primer to mutate yNpl4-3xFLAG Met574 to Glutamine
83	CAAACGTTAGTCATGGCCCTTCAGGAAA GCG	forward primer to mutate yNpl4-3xFLAG Ile575 to Alanine
84	CGCTTTCCTGAAGGGCCATGACTAACGTT TG	reverse primer to mutate yNpl4-3xFLAG Ile575 to Alanine
85	GATGAGCAAGACGGTGCAACCATGGAC GTAG	forward primer to mutate yNpl4-3xFLAG Leu296 to Alanine

86	CTACGTCCATGGTTGCACCGTCTTGCTCATC	reverse primer to mutate yNpl4-3xFLAG Leu296 to Alanine
87	GATTCGTTTTTTCTTTCATCAGCCGAAGTTATTATGGCTGCTAGG	forward primer to mutate yNpl4-3xFLAG Leu353 to Alanine
88	CCTAGCAGCCATAATAACTTCGGCTGATGAAAGAAAAAACGAATC	reverse primer mutate yNpl4-3xFLAG Leu353 to Alanine
89	CCACATTTCCTTCAATGGCAGCCATTAATGACACTACAGATG	forward primer mutate yNpl4-3xFLAG Tyr424 to Alanine
90	CATCTGTAGTGTCATTAATGGCTGCCATTGAAGGAAATGTGG	reverse primer mutate yNpl4-3xFLAG Tyr424 to Alanine
91	CTAGATCTGCCTGAACGCCAATTATTCTTTGG	forward primer to mutate yUfd1-3xFLAG Gly297 to Arginine
92	CCAAAGAATAATTGGCGTTCAGGCAGATCTAG	reverse primer to mutate yUfd1-3xFLAG Gly297 to Arginine
93	CTGCCTGAAGGCCAAGCATTCTTTGGTTTCC	forward primer to mutate yUfd1-3xFLAG Leu299 to Alanine
94	GGAAACCAAAGAATGCTTGGCCTTCAGGCAG	reverse primer to mutate yUfd1-3xFLAG Leu299 to Alanine
95	CTGAAGGCCAATTATTCGCTGGTTTCCCAATGGTC	forward primer to mutate yUfd1-3xFLAG Phe301 to Alanine
96	GACCATTGGGAAACCAGCGAATAATTGGCCTTCAG	reverse primer to mutate yUfd1-3xFLAG Phe301 to Alanine
97	TTTACCTGAGGCGTCAAGCGTTCGCTGGCTTTCCTATGG	forward primer to mutate yUfd1-3xFLAG Leu299 and F301 to Ala-Ala
98	CCATAGGAAAGCCAGCGAACGCTTGACGCCTCAGGTAAA	reverse primer to mutate yUfd1 -3xFLAG Leu299 and F301 to Ala-Ala
99	ACCTGGCAGCTGACCGCCGGATTCCTGAAGGATCA	In-Fusion primer to insert hNpl4 NZF into yNpl4 vector
100	AGCCTGCCACGCACGTAACTCGAGCGGCCGCATCGTGA	In-Fusion primer to insert hNpl4 NZF into yNpl4 vector
101	CTTCAGGAATCCGGCGGTCAGCTGCCAGGTCTGCA	In-Fusion primer to insert hNpl4 NZF into yNpl4 vector
102	ATGCGGCCGCTCGAGTTACGTGCGTGGCAGGCTACA	In-Fusion primer to insert hNpl4 NZF into yNpl4 vector

Supplementary Table 2. Yeast strains used in this study.

Strain name	Genotype (Background)	Source
W303-1A	*MATa ura3-1 trp1-1 leu2-3,112 his3-11,15 ade2-1 can1-100*	Our stock
YHT288	*MATα ura3-1 trp1-1 leu2-3,112 his3-11,15 ade2-1, can1-100 Δnpl4::KanMX* [NPL4-TRP1]	This study
YHT298	*MATα ura3-1 trp1-1 leu2-3,112 his3-11,15 ade2-1, can1-100 Δnpl4::KanMX* [NPL4 WT-3xFLAG-CYC1term -TRP1]	This study
YHT309	*MATα ura3-1 trp1-1 leu2-3,112 his3-11,15 ade2-1, can1-100 Δnpl4::KanMX* [NPL4 A494F-3xFLAG-CYC1term -TRP1]	This study
YHT591	*MATα ura3-1 trp1-1 leu2-3,112 his3-11,15 ade2-1, can1-100 Δnpl4::KanMX* [NPL4 S498R-3xFLAG-CYC1term -TRP1]	This study
YHT311	*MATα ura3-1 trp1-1 leu2-3,112 his3-11,15 ade2-1, can1-100 Δnpl4::KanMX* [NPL4 T571A-3xFLAG-CYC1term -TRP1]	This study
YHT589	*MATα ura3-1 trp1-1 leu2-3,112 his3-11,15 ade2-1, can1-100 Δnpl4::KanMX* [NPL4 M574A-3xFLAG-CYC1term -TRP1]	This study
YHT312	*MATα ura3-1 trp1-1 leu2-3,112 his3-11,15 ade2-1, can1-100 Δnpl4::KanMX* [NPL4 M574Q-3xFLAG-CYC1term -TRP1]	This study
YHT313	*MATα ura3-1 trp1-1 leu2-3,112 his3-11,15 ade2-1, can1-100 Δnpl4::KanMX* [NPL4 I575A-3xFLAG-CYC1term -TRP1]	This study
YHT541	*MATα ura3-1 trp1-1 leu2-3,112 his3-11,15 ade2-1, can1-100 Δnpl4::KanMX* [NPL4 L296A-3xFLAG-CYC1term-TRP1]	This study
YHT542	*MATα ura3-1 trp1-1 leu2-3,112 his3-11,15 ade2-1, can1-100 Δnpl4::KanMX* [NPL4 L353A-3xFLAG-CYC1term-TRP1]	This study
YHT543	*MATα ura3-1 trp1-1 leu2-3,112 his3-11,15 ade2-1, can1-100 Δnpl4::KanMX* [NPL4 Y424A-3xFLAG-CYC1term-TRP1]	This study
YHT544	*MATα ura3-1 trp1-1 leu2-3,112 his3-11,15 ade2-1, can1-100 Δnpl4::KanMX* [NPL4 L296A L353A Y424A-3xFLAG-CYC1term-TRP1]	This study
YHT560	*MATα ura3-1 trp1-1 leu2-3,112 his3-11,15 ade2-1, can1-100 Δufd1::KanMX* [UFD1 WT-TRP1]	This study
YHT555	*MATα ura3-1 trp1-1 leu2-3,112 his3-11,15 ade2-1, can1-100 Δufd1::KanMX* [UFD1 WT-3xFLAG-CYC1term-TRP1]	This study
YHT556	*MATα ura3-1 trp1-1 leu2-3,112 his3-11,15 ade2-1, can1-100 Δufd1::KanMX* [UFD1 G297R-3xFLAG-CYC1term-TRP1]	This study
YHT557	*MATα ura3-1 trp1-1 leu2-3,112 his3-11,15 ade2-1, can1-100 Δufd1::KanMX* [UFD1 L299A-3xFLAG-CYC1term-TRP1]	This study
YHT558	*MATα ura3-1 trp1-1 leu2-3,112 his3-11,15 ade2-1, can1-100 Δufd1::KanMX* [UFD1 F301A-3xFLAG-CYC1term-TRP1]	This study
YHT559	*MATα ura3-1 trp1-1 leu2-3,112 his3-11,15 ade2-1, can1-100 Δufd1::KanMX* [UFD1 G297R L299A F301A-3xFLAG-CYC1term-TRP1]	This study
YHT293	*MATα ura3-1 trp1-1 leu2-3,112 his3-11,15 ade2-1, can1-100 Δnpl4::KanMX* [NPL4 T571A-TRP1]	This study
YHT295	*MATα ura3-1 trp1-1 leu2-3,112 his3-11,15 ade2-1, can1-100 Δnpl4::KanMX* [NPL4 I575A-TRP1]	This study
YHT576	*MATα ura3-1 trp1-1 leu2-3,112 his3-11,15 ade2-1, can1-100 Δnpl4::KanMX* [NPL4 L296A L353A Y424A-TRP1]	This study
YHT577	*MATα ura3-1 trp1-1 leu2-3,112 his3-11,15 ade2-1, can1-100 Δufd1::KanMX* [UFD1 G297R L299A F301A-TRP1]	This study
YHT235	*MATα ura3-1 trp1-1 leu2-3,112 his3-11,15 ade2-1, can1-100 npl4::npl4-1-KanMX*	Ref. 1

1. Tsuchiya, H. et al. Ub-ProT reveals global length and composition of protein ubiquitylation in cells. *Nat. Commun*. 9, 524 (2018)
