# Supplementary material for: Structural insights into ubiquitin recognition and Ufd1 interaction of Npl4
Source: Nat Commun. 2019 Dec 13;10:5708. doi: 10.1038/s41467-019-13697-y (PMC6910952; doi:10.1038/s41467-019-13697-y)
Supplement: Supplementary file 3 — Reporting Summary [file 41467_2019_13697_MOESM3_ESM.pdf]

nature research

Corresponding author(s): Shuya Fukai, Yasushi Saeki

Last updated by author(s): Nov 12, 2019

Reporting Summary

Nature Research wishes to improve the reproducibility of the work that we publish. This form provides structure for consistency and transparency in reporting. For further information on Nature Research policies, see Authors & Referees and the Editorial Policy Checklist .

Statistics

For all statistical analyses, confirm that the following items are present in the figure legend, table legend, main text, or Methods section.

n/a	Confirmed	
☐	☒	The exact sample size (*n*) for each experimental group/condition, given as a discrete number and unit of measurement
☐	☒	A statement on whether measurements were taken from distinct samples or whether the same sample was measured repeatedly
☐	☒	The statistical test(s) used AND whether they are one- or two-sided<br>*Only common tests should be described solely by name; describe more complex techniques in the Methods section.*
☐	☒	A description of all covariates tested
☒	☐	A description of any assumptions or corrections, such as tests of normality and adjustment for multiple comparisons
☒	☐	A full description of the statistical parameters including central tendency (e.g. means) or other basic estimates (e.g. regression coefficient) AND variation (e.g. standard deviation) or associated estimates of uncertainty (e.g. confidence intervals)
☐	☒	For null hypothesis testing, the test statistic (e.g. *F*, *t*, *r*) with confidence intervals, effect sizes, degrees of freedom and *P* value noted<br>*Give P values as exact values whenever suitable.*
☒	☐	For Bayesian analysis, information on the choice of priors and Markov chain Monte Carlo settings
☒	☐	For hierarchical and complex designs, identification of the appropriate level for tests and full reporting of outcomes
☒	☐	Estimates of effect sizes (e.g. Cohen's *d*, Pearson's *r*), indicating how they were calculated

Our web collection on statistics for biologists contains articles on many of the points above.

Software and code

Policy information about availability of computer code

Data collection	Crystallographic data collection: BSS (SPring-8 BL41XU)<br>SPR analysis: Biacore T200 software<br>Fluorescent polarization analysis: EnVision manager
Data analysis	Crystallographic data processing: HKL2000, CCP4, Coot, Phenix<br>SPR analysis: Biacore T200 software<br>Fluorescent polarization analysis: KaleidaGraph<br>Statistical analysis: KaleidaGraph

For manuscripts utilizing custom algorithms or software that are central to the research but not yet described in published literature, software must be made available to editors/reviewers. We strongly encourage code deposition in a community repository (e.g. GitHub). See the Nature Research guidelines for submitting code & software for further information.

Data

Policy information about availability of data

All manuscripts must include a data availability statement. This statement should provide the following information, where applicable:

- Accession codes, unique identifiers, or web links for publicly available datasets
- A list of figures that have associated raw data
- A description of any restrictions on data availability

Coordinates and structure factors of yNpl4, yNpl4–K48-Ub2 and yNpl4–yUfd1 have been deposited in the Protein Data Bank under the accession codes 6JWH, 6JWI, and 6JWJ, respectively. The data that support the findings of this study are available from the corresponding author upon reasonable request.

Field-specific reporting

Please select the one below that is the best fit for your research. If you are not sure, read the appropriate sections before making your selection.

- [x] Life sciences
- [ ] Behavioural & social sciences
- [ ] Ecological, evolutionary & environmental sciences

For a reference copy of the document with all sections, see nature.com/documents/nr-reporting-summary-flat.pdf

Life sciences study design

All studies must disclose on these points even when the disclosure is negative.

Sample size	No sample-size calculations were performed. Sample size was determined to be adequate based on the magnitude and consistency of measurable differences between groups.
Data exclusions	On principle, data were only excluded for failed experiments.
Replication	All attempts at replication were successful.
Randomization	No randomization.
Blinding	n/a

Reporting for specific materials, systems and methods

We require information from authors about some types of materials, experimental systems and methods used in many studies. Here, indicate whether each material, system or method listed is relevant to your study. If you are not sure if a list item applies to your research, read the appropriate section before selecting a response.

Materials & experimental systems

n/a	Involved in the study	
☐	☒	Antibodies
☒	☐	Eukaryotic cell lines
☒	☐	Palaeontology
☒	☐	Animals and other organisms
☒	☐	Human research participants
☒	☐	Clinical data

Methods

n/a	Involved in the study	
☒	☐	ChIP-seq
☒	☐	Flow cytometry
☒	☐	MRI-based neuroimaging

Antibodies

Antibodies used

Primary antibodies
Anti-Ubiquitin, Lys48-Specific, clone Apu2 antibody (Millipore, Cat#: 05-1307, Lot#: 3041800)
Monoclonal ANTI-FLAG M2 Peroxidase (HRP) antibody produced in mouse (Sigma-Aldrich, Cat#A8592; Lot#: SLBH1183V)
Anti-Cdc48 (gifted from Dr. Kimura)
Ub (P4D1) HRP (Santa Cruz Biotechnology, Cat#:sc-8017 HRP, Lot#: D0116)
Phosphoglycerate Kinase Monoclonal Antibody (clone: 22C5D8, Life technologies: Cat#459250, Lot#: #459250)

Secondary antibodies
Peroxidase AffiniPure Rabbit Anti-Mouse IgG + IgM (H+L) (Jackson ImmunoResearch Laboratories: Cat#315-035-048)
Peroxidase AffiniPure Goat Anti-Rabbit IgG (H+L) (Jackson ImmunoResearch Laboratories: Cat#111-035-144)

Validation

All antibodies were used in Western blotting. And these antibodies have been validated to work with Western blotting on the manufacturer's website. Reactivity of Apu2 and P4D1 antibodies against yeast were confirmed by Tsuchiya et al., 2013, 2018, respectively. Anti-phosphoglycerate kinase is validated to perform to yeast on the manufacturer's website. Anti-Cdc48 antibody were validated in Kimura et al., 2009.
